# Supplementary material for: MSCs rescue impaired wound healing in a murine LAD1 model by adaptive responses to low TGF‐β1 levels
Source: EMBO Rep. 2020 Feb 21;21(4):e49115. doi: 10.15252/embr.201949115 (PMC7132342; doi:10.15252/embr.201949115)
Supplement: Supplementary file 1 — Expanded View Figures PDF [file EMBR-21-e49115-s001.pdf]

Expanded View Figures

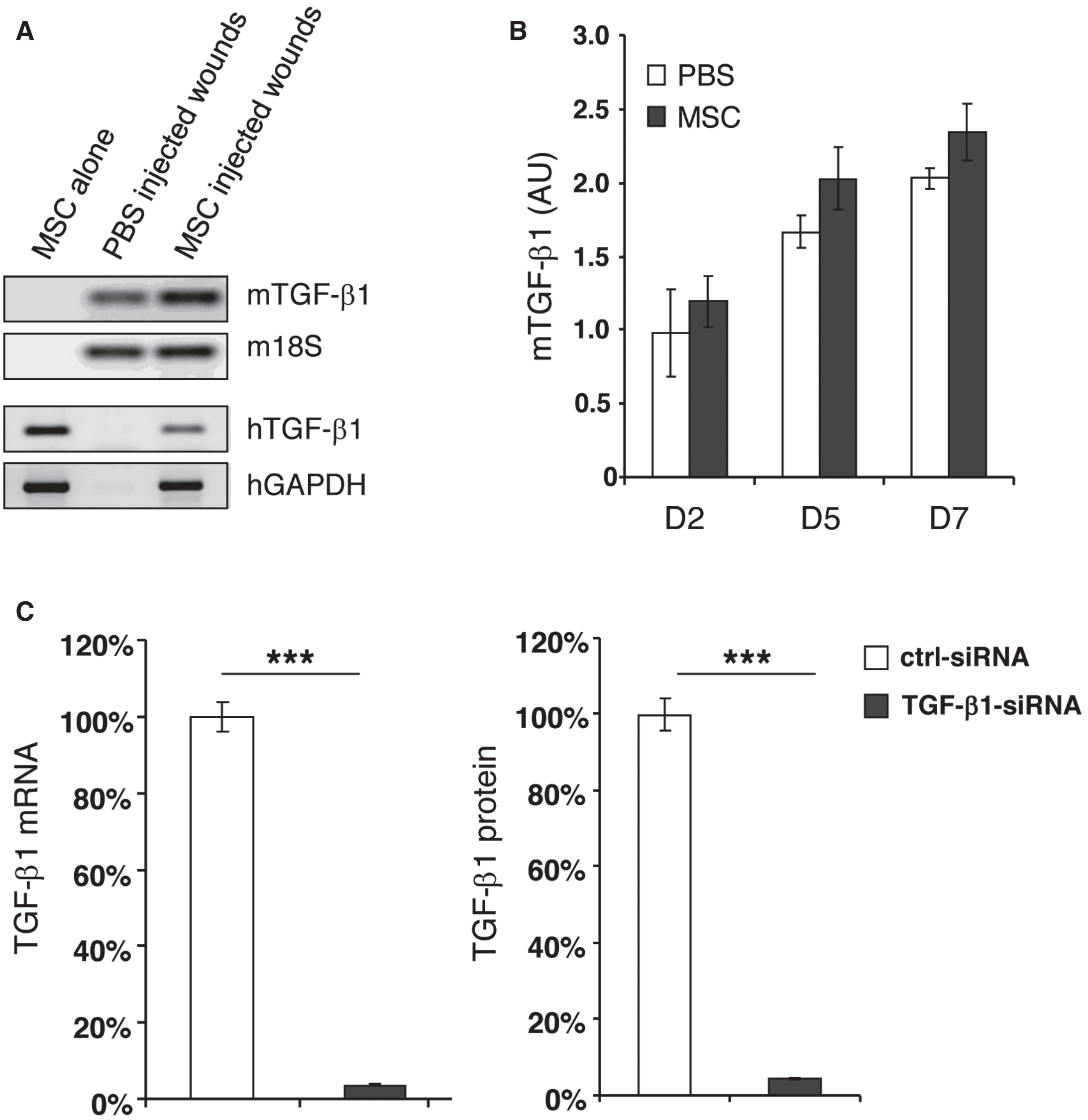

Figure EV1. Quantification of murine- and human-specific TGF-β1 in wounds or in TGF-β1-siRNA-transfected MSCs.

A Total RNA was prepared from cultured AT-MSCs, or day 2 wound tissues from PBS- or AT-MSC-injected CD18$^{-/-}$ wounds. Reverse transcription PCR was performed by using primers that amplify murine- or human-specific TGF-β1. Murine 18S or human GAPDH was used as endogenous control, respectively.

B The expression of murine-specific TGF-β1 in PBS- or AT-MSC-injected CD18$^{-/-}$ wounds at days 2, 5, and 7 post-wounding was quantified by qPCR using primers that amplify murine-specific TGF-β1, and normalized on the expression of murine 18S. Data are given as mean ± SD of triplicate measurements. This experiment was performed three times with similar results. AU, arbitrary unit.

C AT-MSCs were transfected with TGF-β1-specific siRNA targeting four different sites on human TGF-β1 mRNA, and a control siRNA. 48 h after transfection, the cells and supernatants were harvested. The expression of TGF-β1 at mRNA level was quantified by qPCR with primers amplify human-specific TGF-β1. The release of TGF-β1 protein in supernatants was measured by TGF-β1-specific ELISA. The expression and release of TGF-β1 in control siRNA-transfected AT-MSCs was normalized as 100%. The depicted data of TGF-β1-siRNA-transfected AT-MSCs were from the siRNA that showed best knockdown effect among the tested four. This TGF-β1 siRNA was selected for the subsequent experiments. Data are given as mean ± SD of triplicate measurements. ***$P < 0.001$ by two-tailed unpaired *t*-test.

 © 2020 The Authors

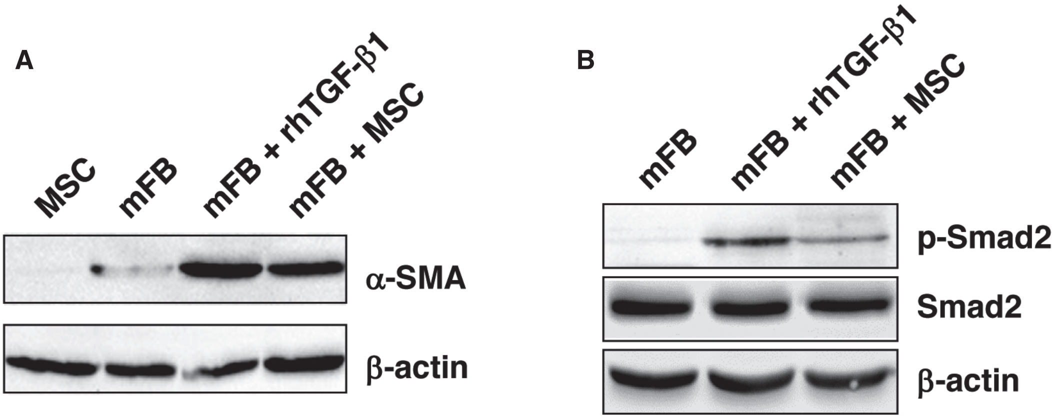

Figure EV2. Expression of α-SMA and phospho-Smad2 in murine fibroblasts cocultured with MSCs.

A, B Protein lysates were prepared from murine primary fibroblasts (mFB) cocultured with human AT-MSCs or cultured alone for 48 h. mFB treated with 2 ng/ml recombinant human TGF-β1 served as positive control. Western blot analysis was performed by using antibodies against α-SMA (A) or total Smad2 and phosphorylated Smad2 (B). β-Actin served as loading control. This experiment was performed twice with similar results.

© 2020 The Authors

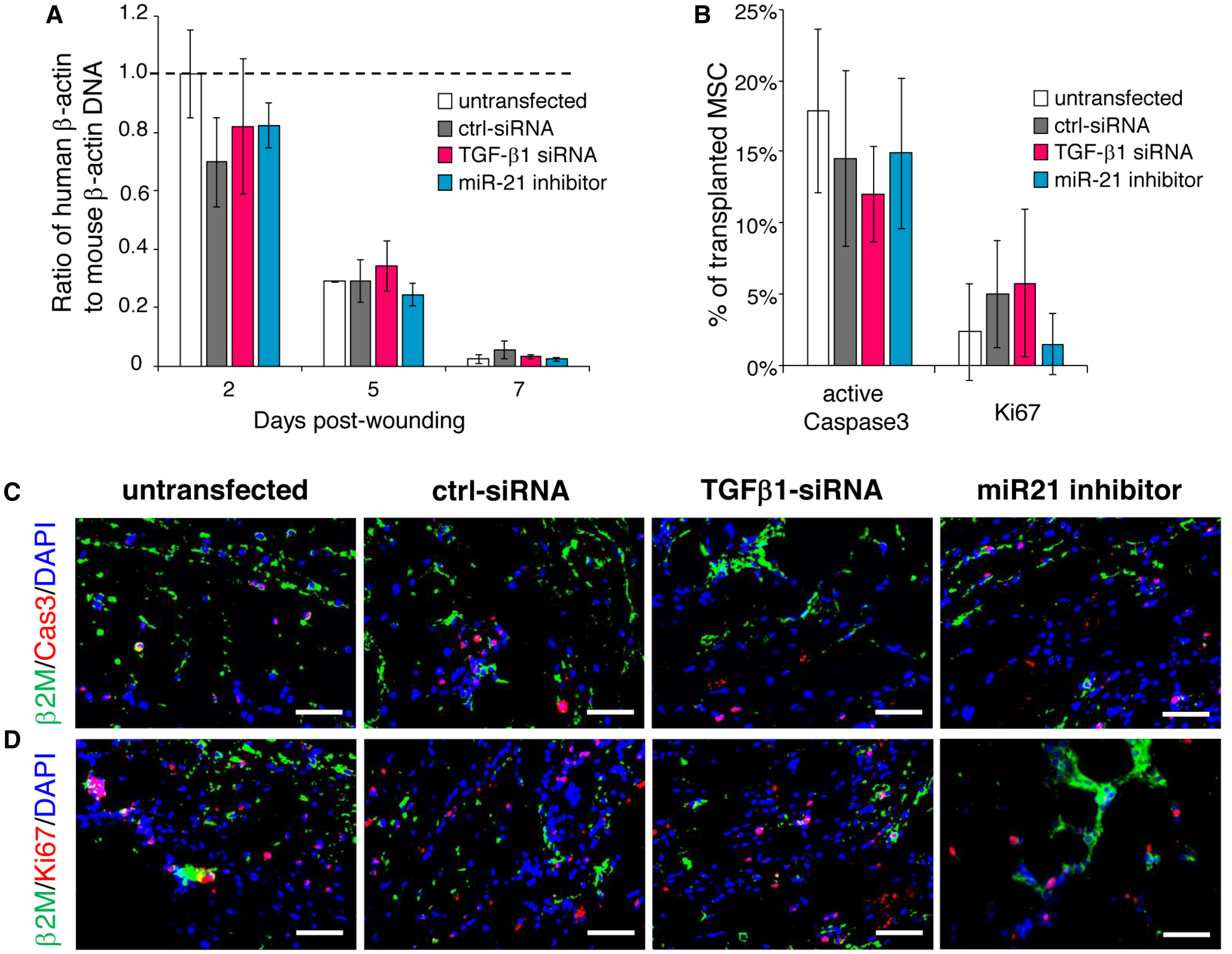

Figure EV3. Transplantation efficiency, survival, and proliferation of TGF-β1-silenced or miR-21-silenced AT-MSCs in CD18$^{-/-}$ wounds.

A TGF-β1-siRNA-transfected MSCs or miR21 inhibitor-transfected MSCs were intradermally injected to CD18$^{-/-}$ wounds at 2.5×10^5 per wound. The transplantation of untransfected MSCs or control siRNA-transfected MSCs at same numbers served as controls. Wound tissues were harvested at days 2, 5, and 7 post-wounding. Genomic DNA was isolated from wound tissues, and the amount of human β-actin DNA was quantified by qPCR normalized on mouse β-actin. Mean ± SD, $n = 3$ wounds per group, not significant by one-way ANOVA with Tukey's test.

B Quantification of immunofluorescence of active caspase 3 (C) and Ki67 (D). Mean ± SD, $n = 3$ wounds per group, not significant by one-way ANOVA with Tukey's test.

C, D Representative immunofluorescence images of active caspase 3 (C) or Ki67 (D) (red) together with human β2M (green). Nuclei were counterstained with DAPI. Scale bars: 50 μm.

 © 2020 The Authors

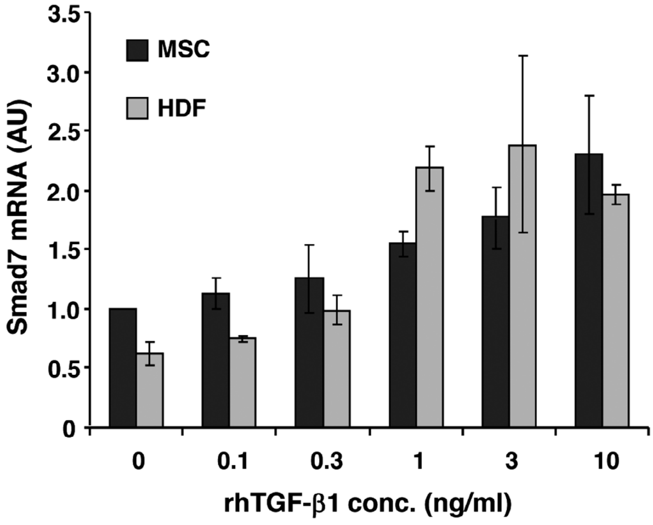

Figure EV4. Expression of Smad7 mRNA in MSCs and HDFs exposed to increasing concentrations of environmental TGF-β1.

Total RNA was prepared from AT-MSCs or HDFs that was cultured in media containing increasing concentrations of recombinant human TGF-β1 at 0, 0.1, 0.3, 1, 3, and 10 ng/ml for 24 h. The expression of Smad7 at mRNA level was quantified by qPCR using primers that amplify human Smad7, and normalized on the expression of human GAPDH. Data are given as mean ± SEM of three independent experiments. AU, arbitrary unit.

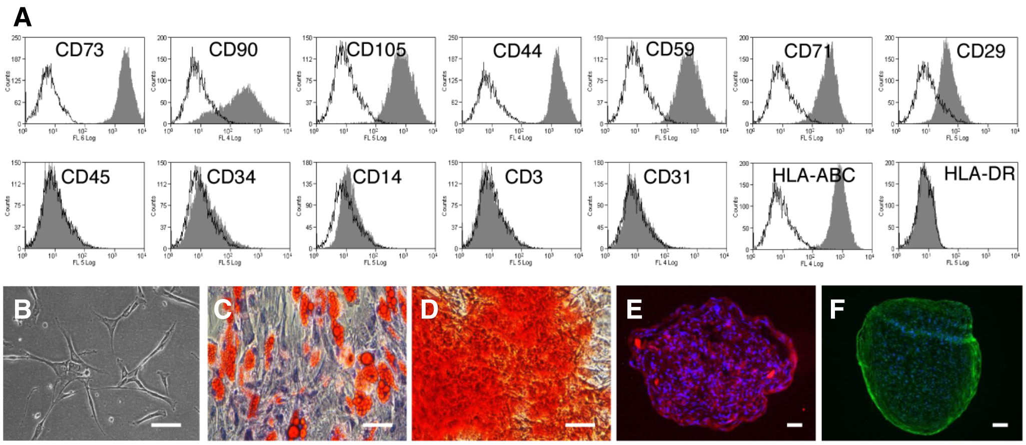

Figure EV5. Characterization of AT-MSCs.

A Flow cytometric analysis of cell surface marker expression on AT-MSCs, including CD73, CD90, CD105, CD44, CD59, CD71, CD29, CD45, CD34, CD14, CD3, CD31, HLA-ABC, and HLA-DR. Marker-specific antibodies labeled AT-MSCs were shown in filled histograms, and the isotype control antibodies labeled cells were shown in empty histograms.
B AT-MSCs are plastic adherent in culture.
C Adipogenic differentiation with Oil Red O staining.
D Osteogenic differentiation with Alizarin Red S staining.
E, F Chondrogenic differentiation with immunostaining of collagen II (E) and aggrecan (F).

Data information: Scale bars (B–F): 100 μm.

© 2020 The Authors
